# Ameliorating the stability of erbium-doped fiber laser using saturable absorber fabricated by the pulsed laser deposition technique

**DOI:** 10.1038/s41598-022-23511-3

**Published:** 2022-11-24

**Authors:** Haroon Asghar, Rizwan Ahmed, Rizwan Ajmal, Zeshan A. Umar, John. G. McInerney, M. Aslam Baig

**Affiliations:** 1 National Centre for Physics, Quaid-I-Azam University Campus, Islamabad, 45320 Pakistan; 2 Department of Physics and Tyndall National Institute, University College Cork, Western Road, Cork, Ireland

**Keywords:** Optics and photonics, Physics

## Abstract

In this paper, we present the performance and stability of an erbium-doped fiber laser (EDFL) based on ZnO saturable-absorber (SA) prepared using two schemes: solution method (SM) and pulsed laser deposition technique (PLDT). It was observed that EDFL with ZnO-SA prepared using SM emits at 1561.25 nm under a pump power of 230 mW. As the pump power is increased from 22.2 mW to 75.3 mW, the pulse duration decreases from 24.91 to 10.69 µs, and the pulse repetition rates increase from 11.59 to 40.91 kHz. Besides at pump power of 75.3 mW, the peak power, pulse energy, and average output power are measured as 0.327 mW, 2.86 nJ, and 0.18 mW, respectively. However, when PLDT-based SA was incorporated into the ring cavity, the emission wavelength is noticed at 1568.21 nm at a pump power of 230 mW. With the increase in pump power from 22.2 mW to 418 mW, the pulse repetition rates increase from 10.79 to 79.37 kHz and the pulse width decreases from 23.58 to 5.6 µs. Furthermore, the peak power, pulse energy, and average output power are observed to be 10.9 mW, 74 nJ, and 5.35 mW, respectively. The stability of EDFL based on SAs prepared using SM and PLDT has also been investigated. To the best of the author's knowledge, it is the first comparison of performance and long-term stability of EDFL based on two experimental techniques SM and PLDT-based SAs. These findings suggest that PLDT-based SAs provides optimum stability over a long period and enhanced the performance of fiber lasers compared to the SAs prepared using the conventional SM technique. This study paves the way for the development of ultra-stable SAs for their potential applications in pulsed laser sources and photonic devices.

## Introduction

Pulsed fiber lasers have attracted much attention in recent years due to their potential applications in spectroscopy, material processing, micro-machining, medical, and telecommunications^1–3^. For pulse formation in lasers, a saturable absorber (SA) is inserted in the cavity which modulates the optical losses that have major applications in Q-switching and mode-locking of lasers. Hence, SA is a key component for achieving ultra-short pulse operation from fiber lasers. A variety of SAs, such as carbon nanotube^4,5^, graphene^6^, Oxide films based SAs^7,8^, semiconductor saturable-absorber mirrors (SESAMs)^9,10^, and topological insulators^11,12^ have been implemented in fiber lasers and cavities for the passive mode-locked pulse generation. Among oxide films, ZnO material is considered a viable material due to its electrical and optical characteristics. ZnO has a direct band gap of 3.37 eV^13^, optimum thermal, chemical, and mechanical stability, low threshold voltage, and ultrafast recovery time^14–17^. Due to these interesting characteristics, ZnO has potential applications in short-wavelength optoelectronic devices, ultraviolet (UV) laser diodes, and light-emitting diodes^18^. Most recently, ZnO-based SAs in erbium/ytterbium doped fiber lasers has attracted much attention from researchers. The fundamental characteristics of an ideal SA are its long-term stability, high damage threshold, fast recovery time, low saturation intensity, optimum modulation depth, and easiness of fabrication and implementation in the laser cavity. The complicated optical alignment, stability, complex fabrication processes, and environmental sensitivity restrict practical applications of SAs for Q-switching and mode-locking operation. Many experimental techniques such as deposition of nanoparticles on a fiber ferrule^19,20^, solution method (SM)^21–23^, and pulsed laser deposition technique (PLDT)^24,25^ have been proposed and demonstrated to fabricate SAs in laser cavities for Q-switching and mode-locking of optical pulses. However, the SAs prepared using conventional techniques like SM and nanoparticles-based techniques are highly unstable and difficult to align inside the laser cavity as they are environmentally sensitive and have a low damage threshold. In the literature, the short-term stability of EDFL has been reported and the output power of optical spectra was measured for 30–60 min^26–29^. The short-term timing stability limits the practical applications of pulsed fiber lasers where a constant and stable pulse operation is required over a long time. To address this challenge, we first measured the stability of our proposed EDFL in terms of peak-to-peak voltage (V_P–P_) of output pulse operation for continuously 5 h. Besides a comparison of various proposed experimental techniques is highly desired to identify the best approach for the fabrication of highly stable SAs for fiber lasers which are easy to align and provide a high damage threshold inside the laser cavities.

In this paper, we compared the performance and stability of Q-switched EDFL based on ZnO-SA prepared using SM and PLDT. The technique which results in the best performance, optimum stability, and high damage threshold was identified. This study suggests that EDFL based on SA fabricated using PLDT yields the narrowest pulse duration, higher repetition rates, and high average output power compared to SA prepared using the SM technique. Besides, it is inferred that PLDT-based SAs provides a large damage threshold and ultra-stable output pulse operation over a long time relative to that prepared using the SM technique. In contrast to the earlier reports regarding the stability of EDFL, the (V_P–P_) stability of pulsed operation has been discussed.

## Fabrication and characterization of Q-switched erbium-doped fiber laser

### Preparation of ZnO-SA using a pulsed laser deposition technique

In the present work, the ZnO thin film is directly deposited on a fiber ferrule using a PLD system. The interaction between the laser and the target governs the deposition process. A high-energy laser beam is focused on a target material inside the PLD chamber. As the laser beam hits the target, a visible plasma plume is formed which expands in the surroundings according to the principle of thermodynamics and deposits on a substrate in one or more crystallographic orientations. Laser ablation is the most attractive part of this technique as it maintains the stoichiometry of the target material. By keeping the target and substrate relatively at rest, a non-uniform film is formed as it spreads perpendicular to the substrate. However, to get a uniform thin film, both target and substrate are rotated in opposite directions relative to each other. The PLD technique is mostly recognized for the fabrication of hetero-structures, but now it can be used to control and grow nano-sized thin films.

**Figure 1:**
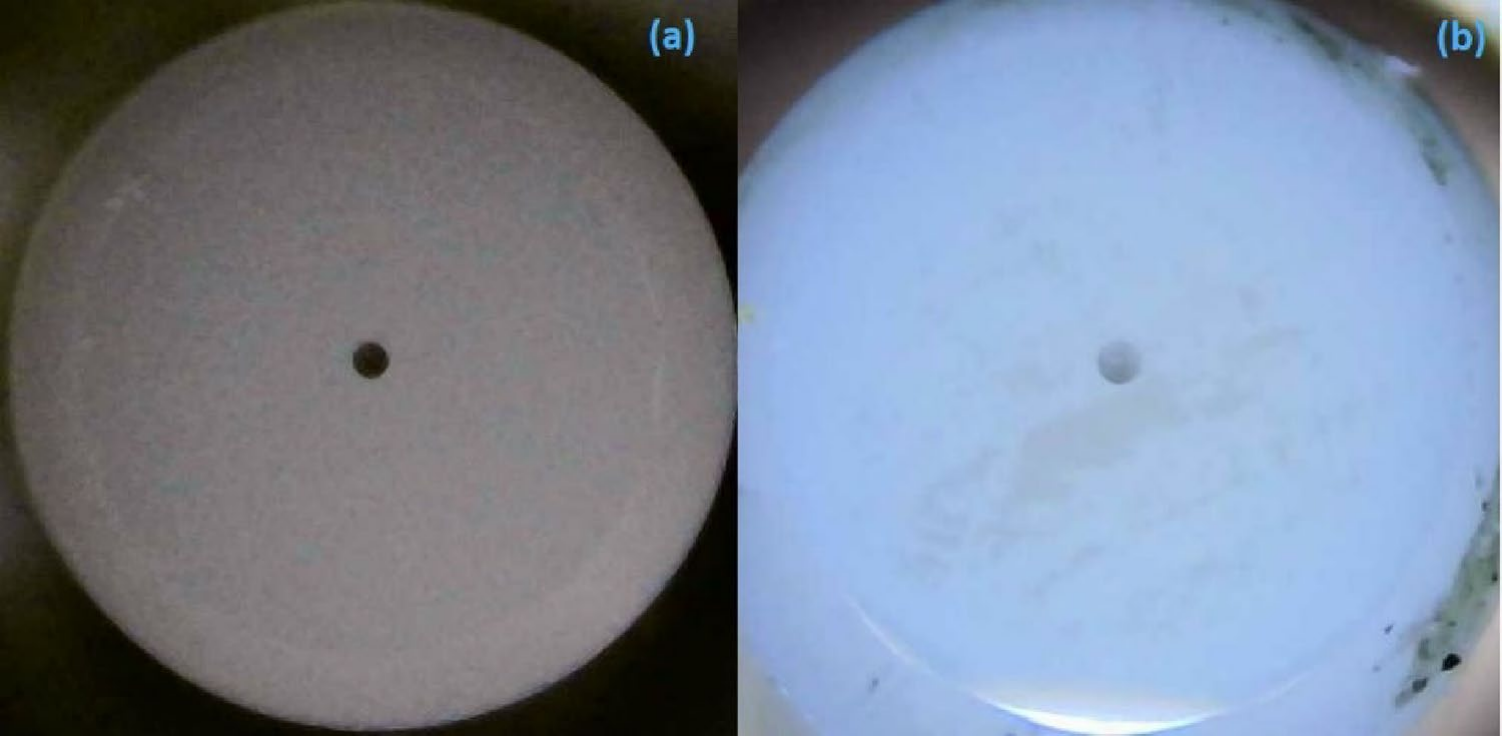
Fiber ferrule (**a**) without SA and (**b**) with ZnO thin film deposit using PLDT.

Here, the fourth harmonic of an Nd:YAG laser emitting at 266 nm was used for the ablation of the ZnO target. A laser beam of 10 mJ was focused on the target inside the PLD chamber and the target (ZnO) was rotated continuously for avoiding the creation of a crater. The Fiber ferrule was installed in front of the target at a distance of 3 cm. The deposition was performed at the rate of 0.05 nm/s and the film thickness was monitored using a quartz crystal microbalance (QCM) which has been already validated and calibrated using the cross-sectional SEM^30^. The substrate (Fiber ferrule) temperature was kept at 25 °C with a background vacuum of 2 × 10^–6^ mbar. Using the PLD technique, a 400 nm thick thin film of ZnO was deposited on the fiber ferrule directly. In Fig. 1a,b, a fiber ferrule is shown without any SA deposited on a fiber ferrule and with ZnO thin film deposited using PLDT, respectively. These images were recorded using the digital microscope (Inskam).

### Preparation of ZnO thin film using solution method technique

**Figure 2:**
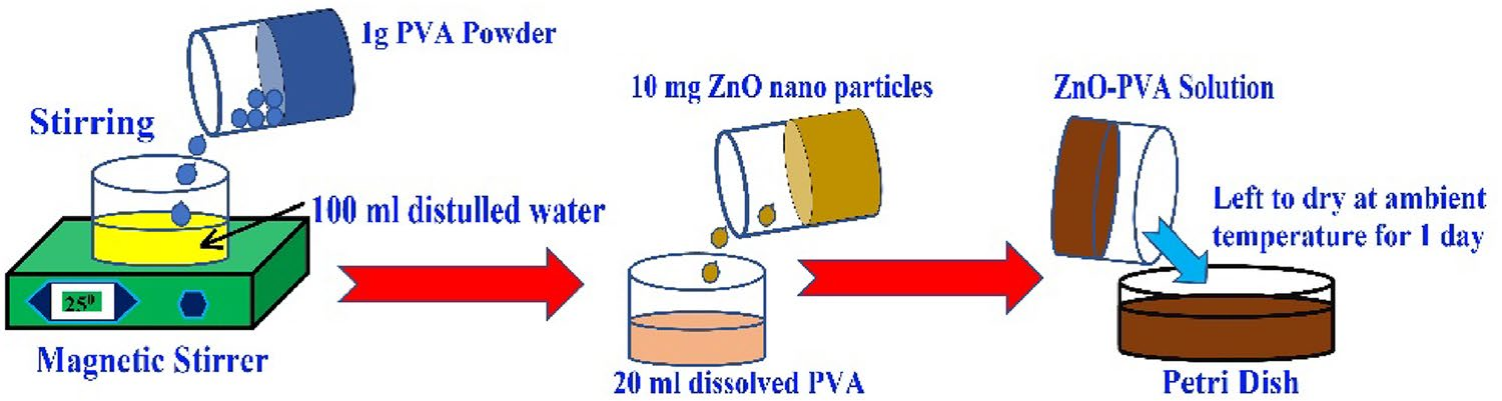
The fabrication process of ZnO-PVA thin-film using SM technique.

**Figure 3:**
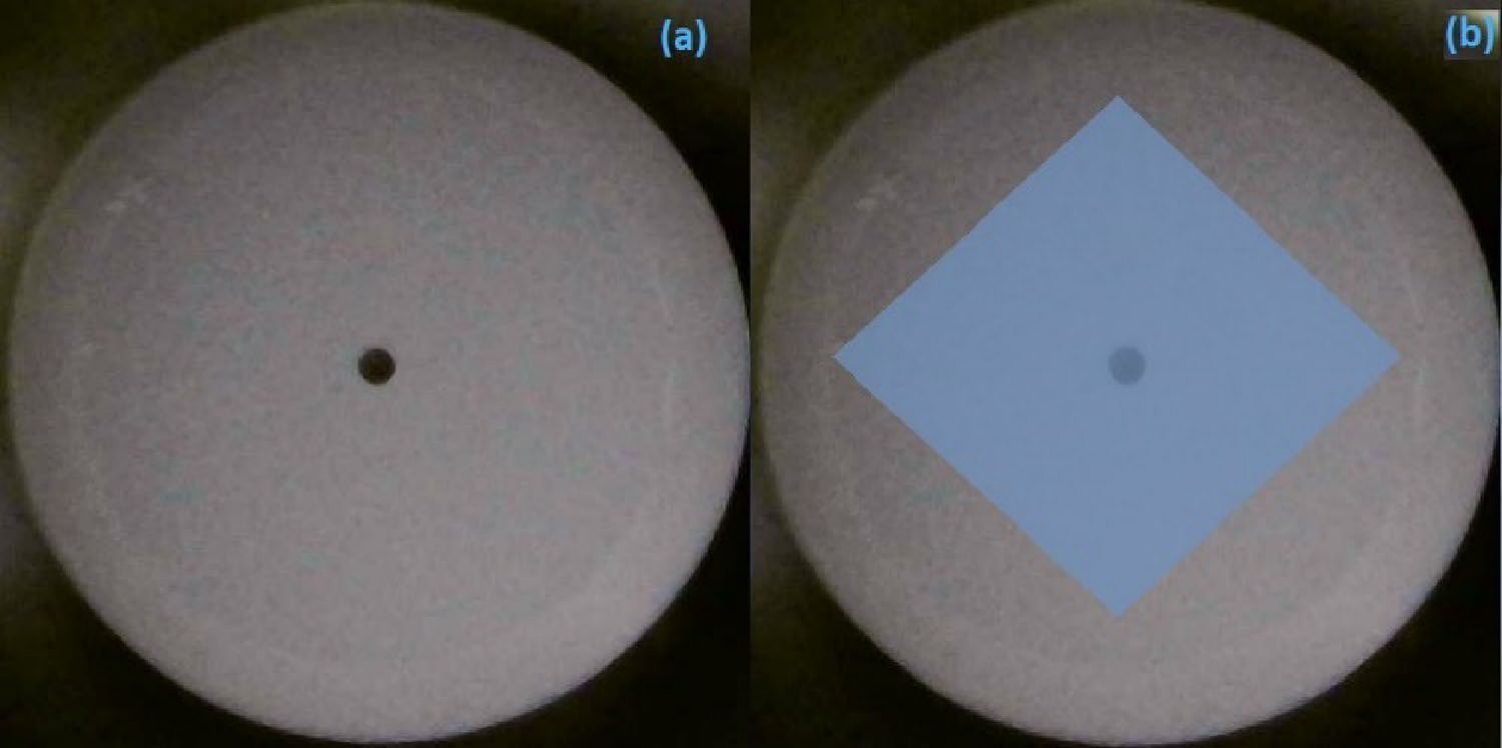
(**a**) Fiber ferrule without thin film deposited and (**b**) with ZnO thin film deposit using SM technique.

The fabrication process of the ZnO thin-film is depicted in Fig. 2. To integrate the ZnO thin film-based SA inside the laser cavity, the ZnO nanostructures were embedded into the polymer-based thin film. The ZnO nanoparticles were purchased from Sigma-Aldrich (USA) and the size of the ZnO particles was estimated in the range of ≤ 50 nm. First, the polyvinyl alcohol (PVA) as the host polymer was prepared by adding 1 g of PVA powder into 100 ml of distilled water. To completely dissolve PVA into the distilled water, the mixture was magnetically stirred at the temperature of 25 °C. After that, 10 mg of ZnO nanoparticles were added to 20 ml of the dissolved PVA suspension. Finally, the ZnO-PVA solution was poured into a petri dish and was left to dry for 1 day at an ambient temperature for the development of the thin film to be used as passive SA. Then a small area of the film was cut and then attached to the surface of the fiber ferrule. The fiber ferrule without-SA and with SA thin film is shown in Fig. 3a,b, respectively.

### Characterization of ZnO-SA

**Figure 4:**
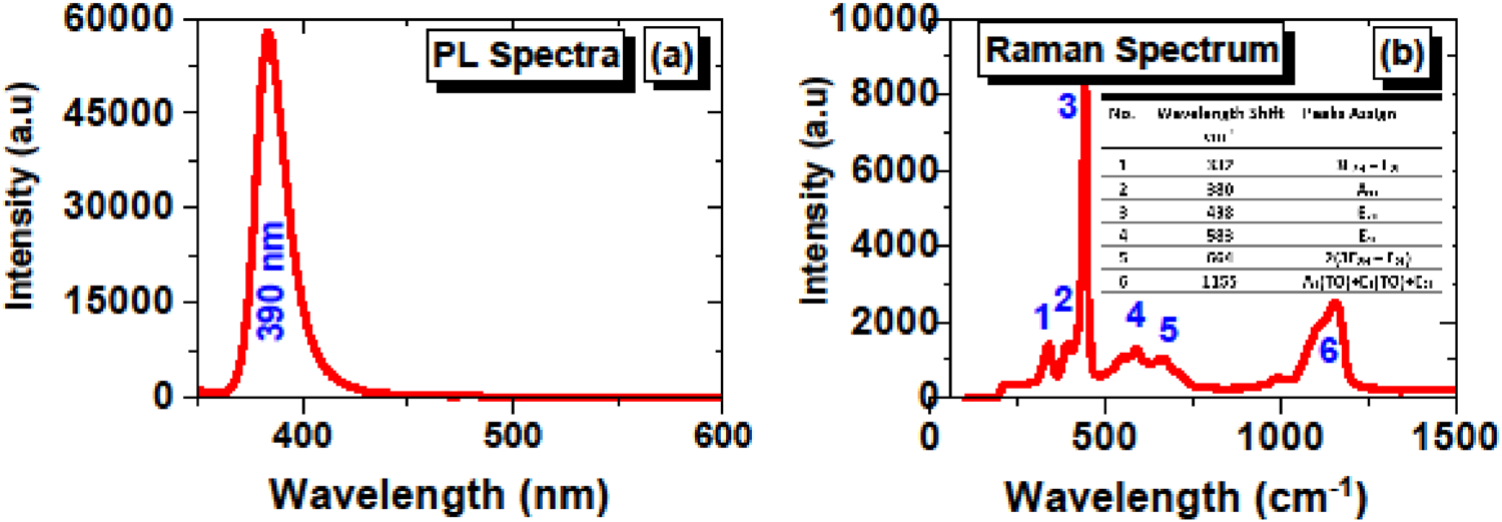
(**a**) PL and (**b**) Raman spectra of ZnO thin film.

The room temperature photoluminescence (PL) spectrum of the deposited ZnO thin film is shown in Fig. 4a. A beam of the nitrogen laser (NL100) emitting at wavelength 337 nm, with 170 µJ pulse energy and a 3.5 ns of pulse duration, was used as the excitation source for photoluminescence. The peak appearing at around 390 nm is correlated with the free exciton recombination or exciton-exciton collisions termed NBE (near band edge) UV emission.

The Raman spectroscopy of the deposited ZnO thin film was done using the Ava Raman with 532 nm laser with maximum power 50 mW. Figure 4b shows the Raman spectrum of the ZnO thin film deposited on the tip of fiber ferrule. The spectroscopic peaks with wavelength shift and their corresponding phonon modes have been assigned as reported in literature^31,32^ and are listed in inset of Fig. 4b.

**Figure 5:**
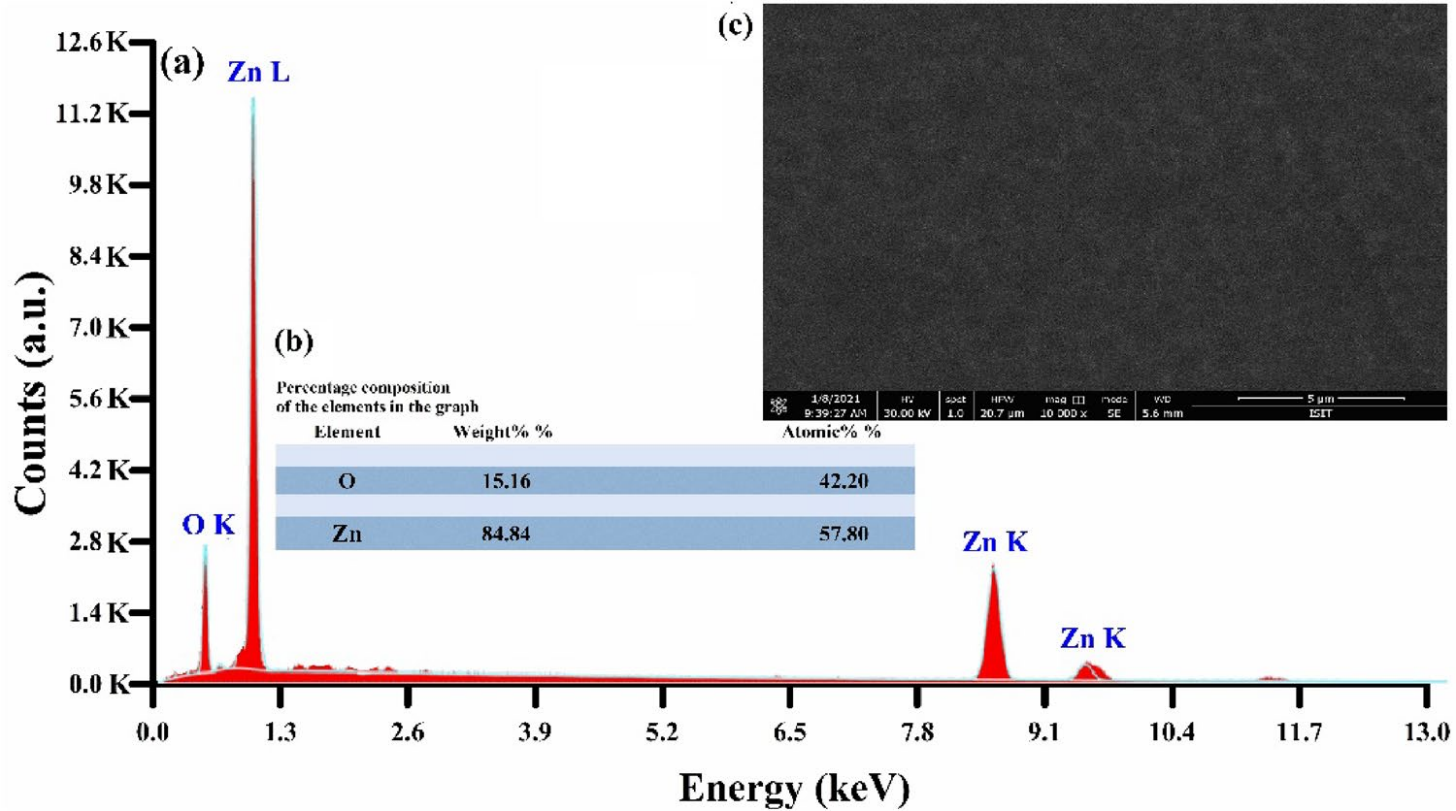
(**a**) Energy dispersive X-ray spectrum of the ZnO thin-film (**b**) Percentage composition of elements present in the ZnO thin-film (**c**) the microphotograph of the surface of the ZnO thin-film.

The EDX spectra of ZnO thin film is shown in Fig. 5. The main elements and the percentage composition present in the ZnO thin film are listed in Fig. 5b. The surface morphology of the ZnO thin film was studied using a field emission scanning electron microscopy (FESEM). The micrograph confirms that the deposited thin film is very smooth in general, and continuous as shown in Fig. 5c. The measured results demonstrate that ZnO thin film has good purity, including Zinc—84.84 wt.% and Oxygen—15.16 wt.%.

## Experimental setup

**Figure 6:**
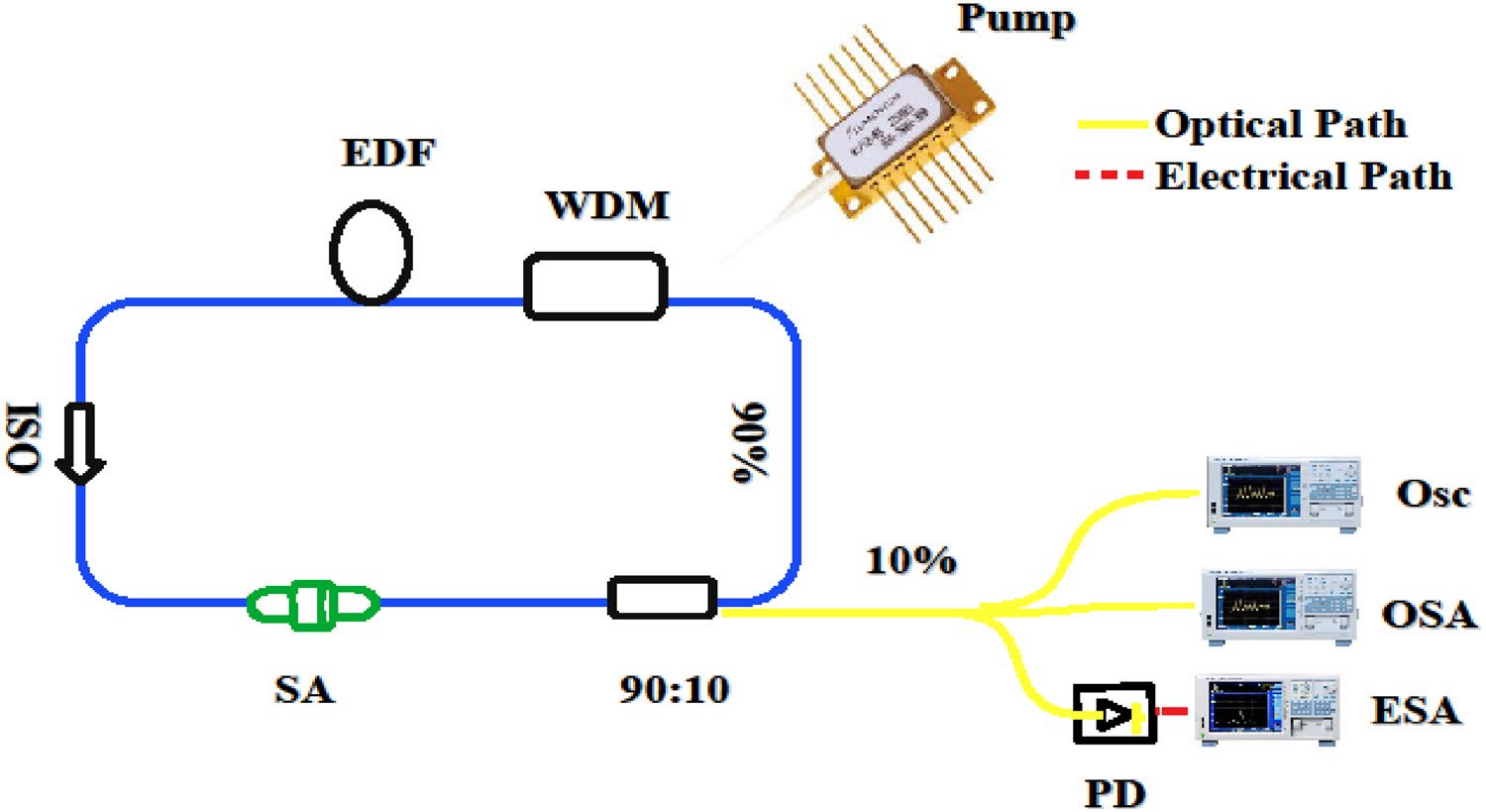
Experimental arrangements of Q-switched erbium-doped fiber laser based on ZnO-SA; *WDM* Wavelength Division Multiplexer, *EDF* Erbium-doped fiber, *ISO* Optical isolator, *PD* Photodiode, *PM* Power meter, *ZnO-SA* Zinc-oxide saturable absorber, *Osc* Oscilloscope, *OSA* Optical spectrum analyzer, *ESA* Electric spectrum analyzer.

Figure 6 shows the schematic diagram of the Q-switched EDFL setup used in the present study. A single mode diode laser emitting at 976 nm was used as a pump source. The output of the pumped laser was coupled with a 980/1550 nm fused wavelength-division multiplexer (WDM) and the common port was coupled with EDF. A polarization independent isolator (PI-ISO) was used to ensure the unidirectional light propagation in the ring cavity. After the isolator, ZnO-SA was incorporated into the laser cavity. Subsequently, a 90:10 output coupler was used to splits the light into two parts; 90% propagates into the ring cavity and 10% was used for analysis. The output power was measured using an optical power meter (Thorlabs). The RF spectra were recorded using an RF spectrum analyzer (GW INSTEK, GSP-9330) via a 5 GHz InGaAs photodiode (Thorlabs: DET08CFC/M). The optical spectra were registered through an optical spectrum analyzer (YOKOGAWA, AQ6370D) with a minimum resolution of 0.02 nm that covers wavelength range from 600 to 1700 nm. A digital oscilloscope (GW INSTEK, GDS-3504) was also used through a 5 GHz InGaAs photodiode (Thorlabs: DET08CFC/M) for analyzing the pulse train properties.

## Results and discussions

### Q-switched performance of EDFL based on ZnO-SA

**Figure 7:**
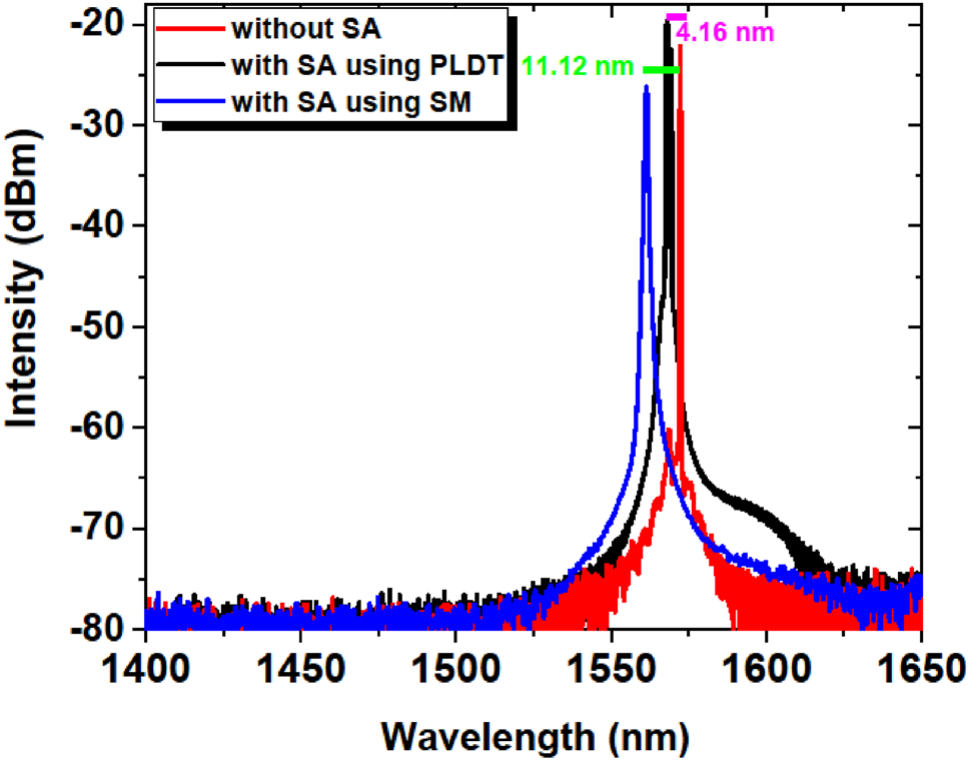
Measured optical spectrum of EDFL without SA (solid red line) and for SA prepared using SM (solid blue line) and PLDT (solid black line).

A CW operation of a Q-switched EDFL is observed at a low pump threshold of 11.2 mW. However, when SM and PLDT-based ZnO-SA is incorporated into the laser cavity, the passive Q-switched pulse operation is observed at 22.2 mW. Figure 7 shows the typical optical spectrum of a Q-switched EDFL in continuous mode (solid red line), with ZnO-SA, prepared using SM (solid blue line) and PLDT (solid black line) at pump power 230 mW. The 3-dB bandwidth of the lasing wavelength spectra without SA is 0.4 nm with a center wavelength of 1572.37 nm. However, for SA prepared using SM the 3-dB bandwidth is 1.7 nm at the center wavelength of 1561.25 nm and 1.1 nm for SA fabricated using PLDT at 1568.21 nm central wavelength. This broadening in the optical spectrum from 0.4 nm to 1.1 and 1.7 nm indicates the change in laser behavior to pulse operation from CW mode because it required more Fourier spectral components^33,34^. Besides, a blue shift of 11.12 nm and 4.16 nm in the center wavelength is observed when the SA prepared using SM and PLDT is inserted into the laser cavity, respectively. Due to the insertion of SA inside the laser cavity high optical losses occur and to overcome these losses more gain is acquired, hence altering the wavelength towards the shorter wavelength region.

**Figure 8:**
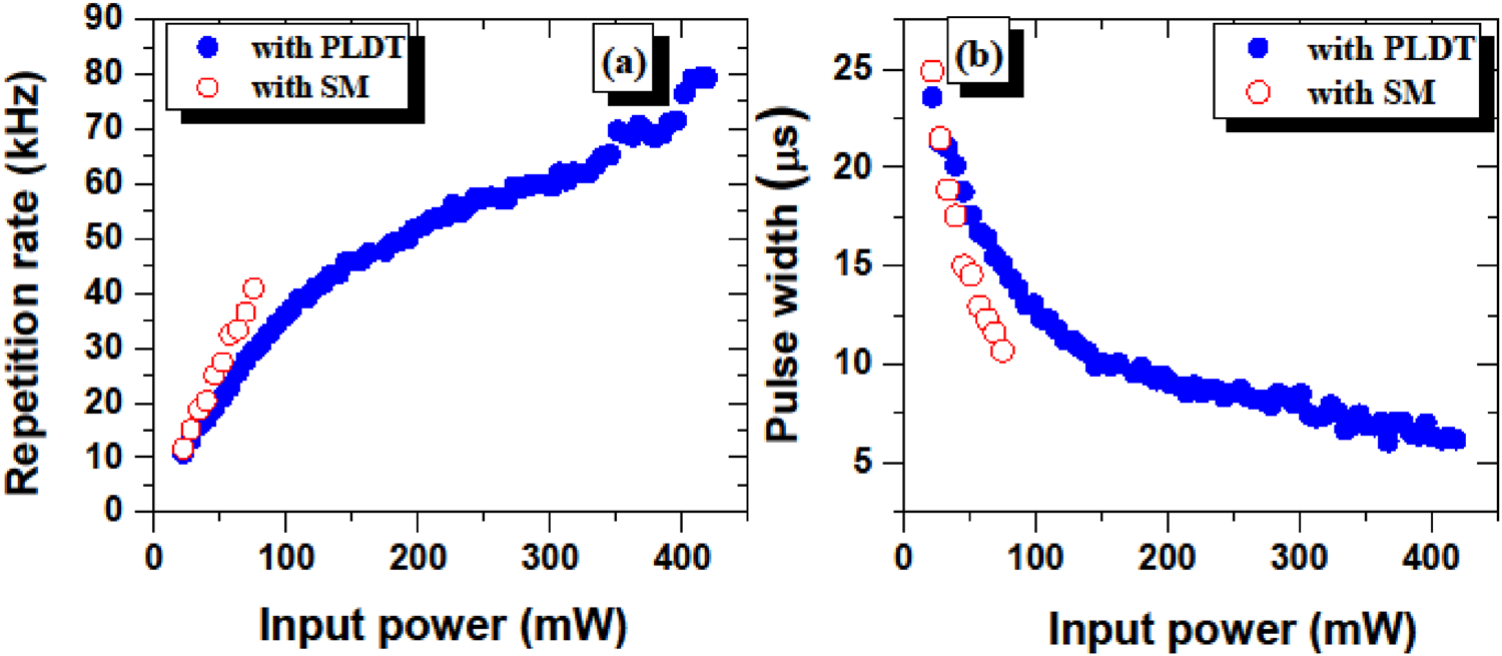
Measured (**a**) pulse repetition rates for SA prepared using SM (hollow red circles) and PLDT (solid blue circles) and (**b**) pulse width for SA prepared using SM (hollow red circles) and PLDT (solid blue circles) as a function of pump power.

The pulse repetition rate and pulse duration as a function of pump power ranging from 22.2 to 418 mW are presented in Fig. 8a,b, respectively for the SA prepared using PLDT (solid blue circles). Similarly, under pump power 22.2–75.3 mW, the pulse repetition rate and the pulse width data are shown in Fig. 8a,b for SA prepared using the SM technique.

These results demonstrate that with SA prepared using SM and PLDT, the Q-switching operation starts at 22.2 mW pump power. For SA fabricated using PLDT, as the pump power is increased from 22.2 to 418 mW, the pulse repetition rates increase from 10.79 to 79.37 kHz, while the pulse duration decreases from 23.58 to 5.6 µs. On the other hand, with SA prepared using the SM technique, an increase in the pump power from 22.2 to 75.3 mW leads to an increase in the pulse repetition rates from 11.59 to 40.91 kHz, while pulse duration decreases from 24.91 to 10.69 µs. With a further increase in the pump power up to 75.3 mW and 418 mW for SA prepared using SM and PLDT, respectively, the Q-switched operation disappears and a continuous-wave operation appears.

**Figure 9:**
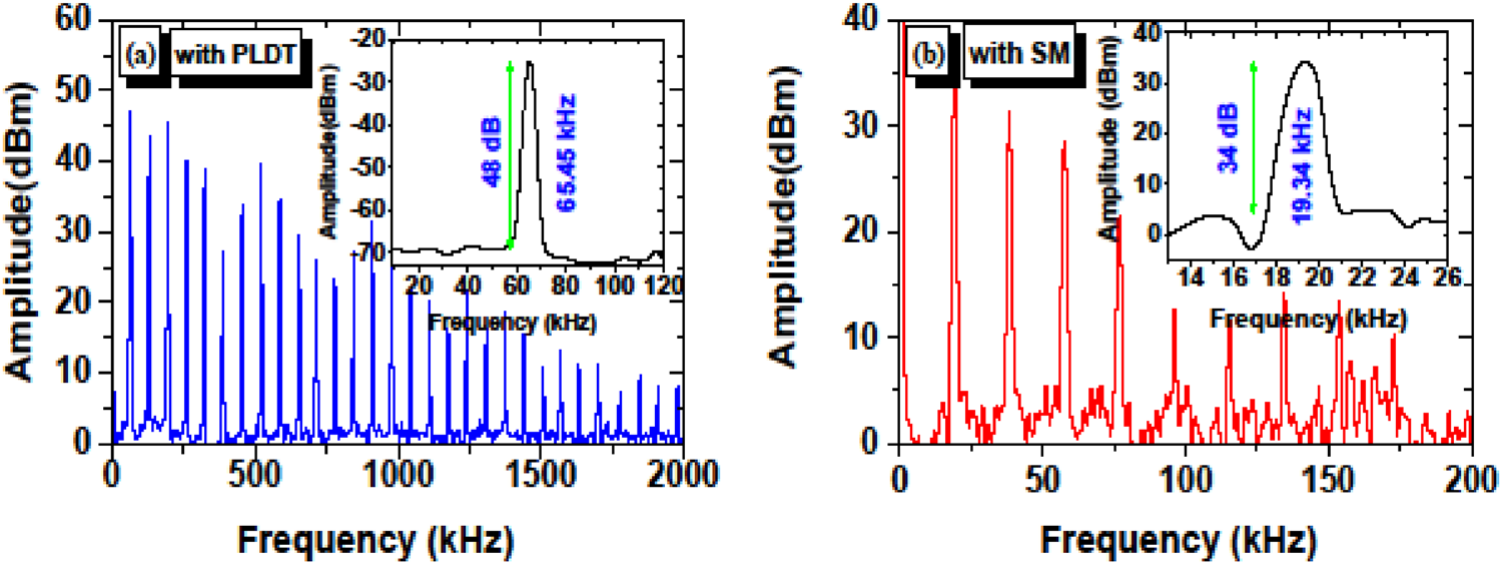
Measured RF spectra for SA prepared using (**a**) PLDT under frequency span 2 MHz, resolution bandwidth 1 kHz and video bandwidth 10 Hz and (**b**) SM technique under frequency span 200 kHz, resolution bandwidth 1 kHz and video bandwidth 10 Hz; inset shows SNR under pump power 45.7 mW.

At a pump power of 283 mW, the RF spectrum is shown in Fig. 9a for SA prepared using PLDT. The RF spectrum was measured using a resolution bandwidth of 1 kHz and a video bandwidth of 10 Hz. From the measured RF spectra for SA prepared using PLDT, 30 frequency harmonics are observed under a frequency span of 2 MHz and the peak of the fundamental frequency is observed at 65.45 kHz. Furthermore, the signal-to-noise ratio (SNR) of the measured RF spectra for SA prepared using PLDT is 48 dB (refer to the inset in Fig. 9a). On the other hand, at a fixed pump power of 75.3 mW, the RF spectrum is shown in Fig. 9b for SA prepared using SM technique. For SA prepared using the SM technique, 10 frequency harmonics are observed under resolution bandwidth of 1 kHz, video bandwidth of 10 Hz, and frequency span of 200 kHz. With SA prepared using the SM technique, the fundamental frequency is observed to be 19.34 kHz with 34 dB SNR. The higher SNR for SA prepared using the PLDT technique further confirms better stability and performance of EDFL based on ZnO-SA prepared using the SM technique.

**Figure 10:**
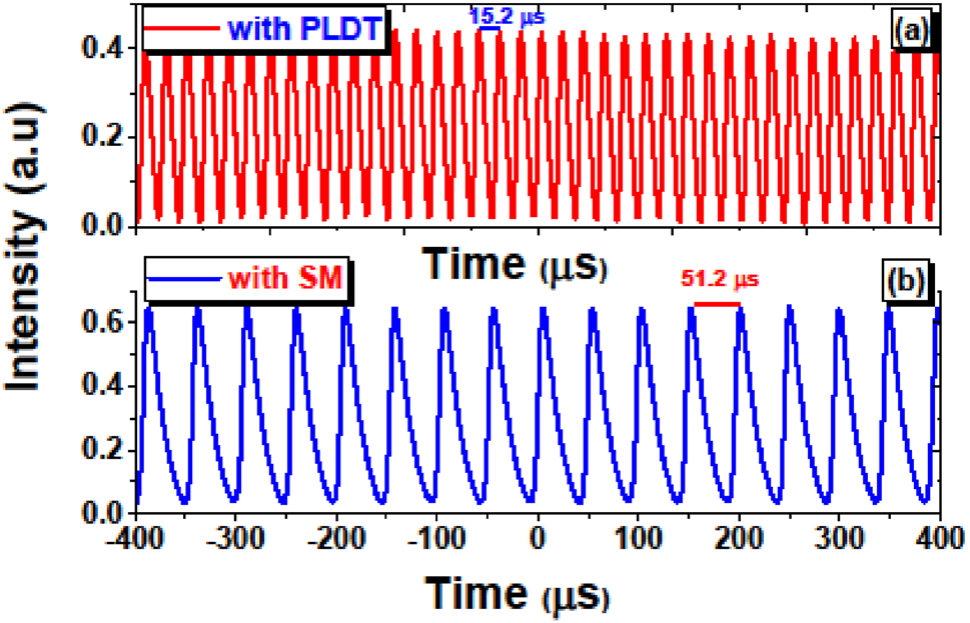
Measured pulse train of EDFL based on SA prepared using (**a**) PLDT at pump power 283 mW (**b**) SM technique at pump power 45.7 mW.

A measured optical pulse train at pump power 283 mW is shown in Fig. 10a for SA prepared using the PLDT and the minimum pulse duration is noticed to be 8.52 µs. The pulse interval under 283 mW pump power is observed to be 15.2 µs which agrees well with the repetition rates of 65.78 kHz. In Fig. 10b the measured optical pulse train is shown for SA fabricated using the SM technique at a pump power of 45.7 mW and the minimum pulse duration is noticed to be 14.99 µs. While, at 45.7 mW pump power, the pulse interval is noticed to be 51.22 µs, which corresponds well with the repetition rates of 19.31 kHz.

It is pertinent to mention here that for SA prepared using the SM technique, as the pump power approaches 75.3 mW, the Q-switching operation of EDFL disappears and a CW operation sets in. However, the SA fabricated with PLDT tolerates up to 418 mW maximum power, and a stable Q-switched pulse operation is still retained. On the other hand, for a pump power higher than 418 mW, the Q-switching disappears and a CW operation commences. This behavior indicates the efficacy of the PLDT over the SM technique for the fabrication of robust, stable, and reliable SAs for fiber lasers.

**Figure 11:**
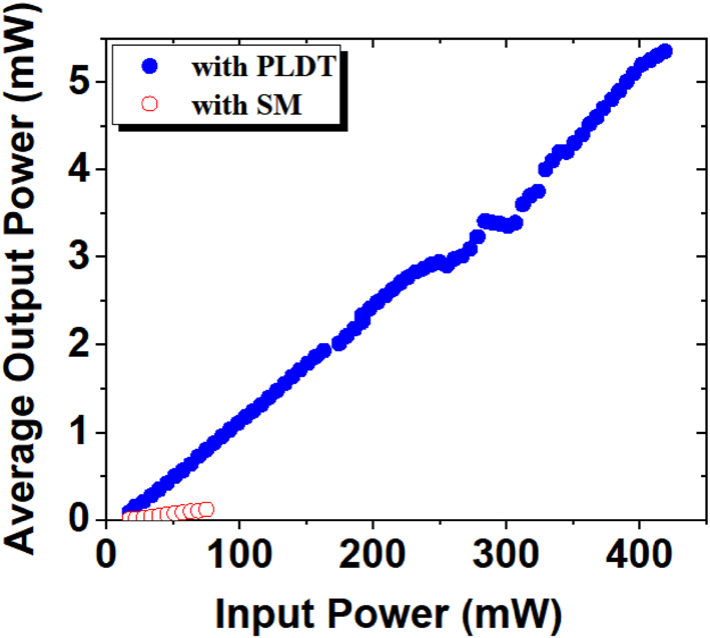
Measured average output power versus pump power for SAs prepared using SM (hollow red circles) and PLDT (solid blue circles).

The average output power of EDFL with both SAs is shown in Fig. 11. For the SM technique, the maximum average output power is 0.18 mW at a pump power of 75.3 mW. With SA prepared using PLDT, 5.35 mW average output power was observed at 418 mW pump power.

**Figure 12:**
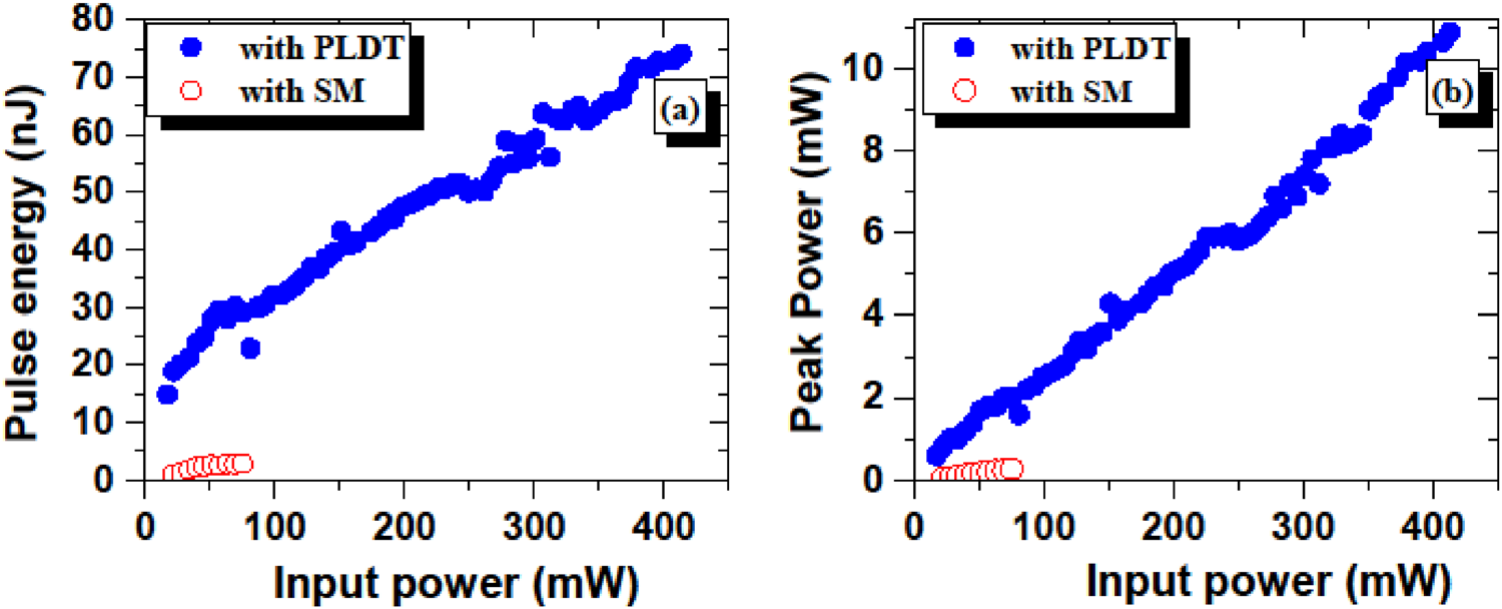
Measured (**a**) pulse energy and (**b**) peak power as a function of pump power for SA prepared using SM (hollow red circles) and PLDT (solid blue circles).

For the SM technique-based SA, the pulse energy and peak power are noted to be 2.86 nJ and 0.327 mW, respectively. The pulse energy of 74 nJ and 10.9 mW peak power is observed at 418 mW pump power with SA prepared using PLDT. The results presented in Figs. 11 and 12, reveal that EDFL based on SAs prepared using PLDT yields higher output power and provides better saturation tolerance than SAs prepared using the SM technique.

### Q-switched performance of EDFL based on ZnO-SA

**Figure 13:**
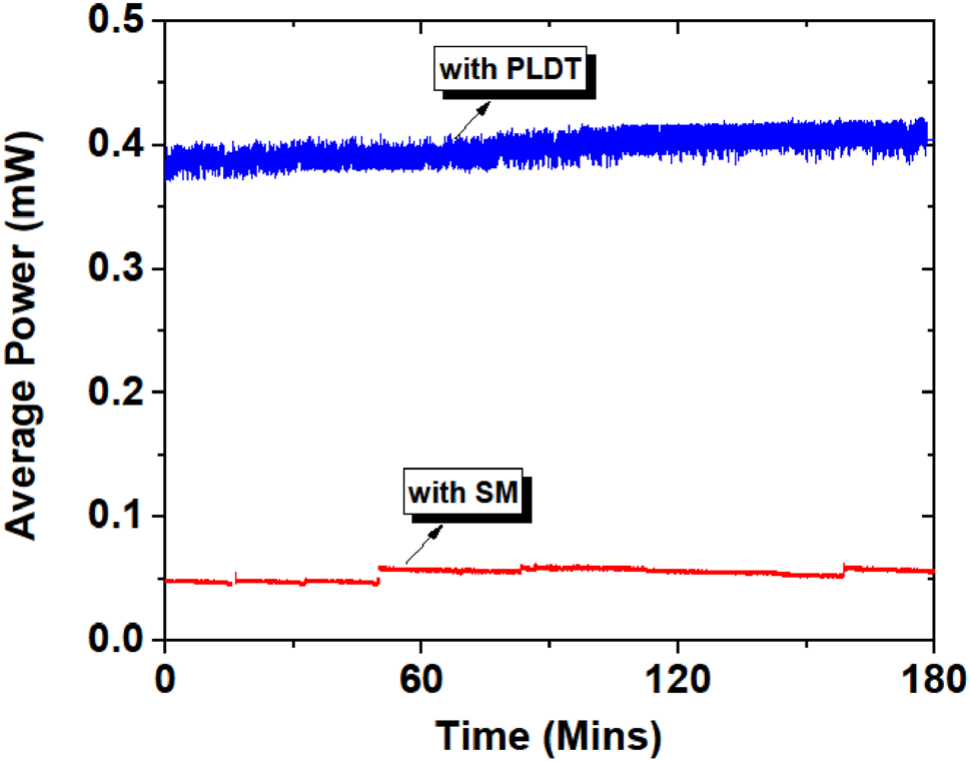
Measured average output power as a function of time for SA prepared using SM and PLDT.

**Figure 14:**
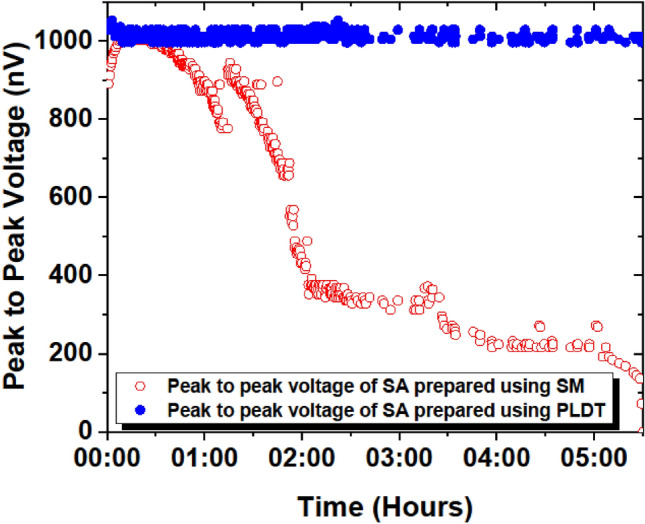
Measured V_P–P_ as a function of time for SA prepared using SM (hollow red circles) and PLDT (solid blue circles).

**Figure 15:**
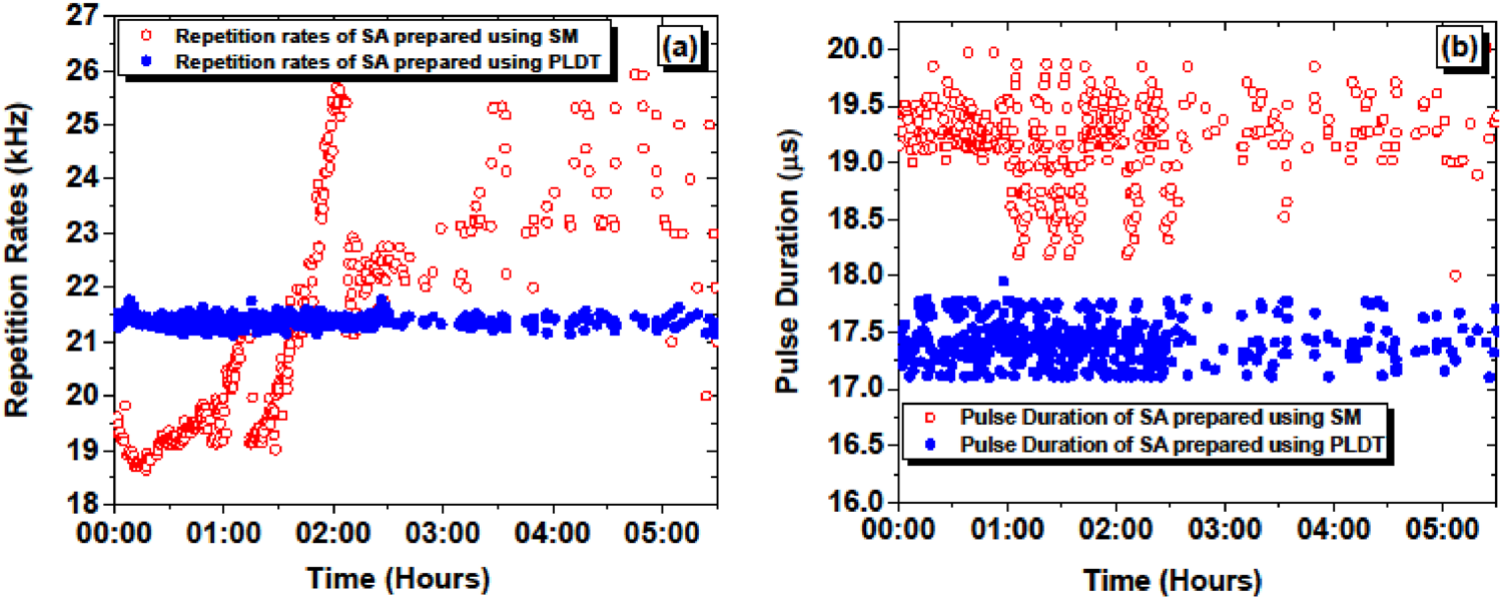
Measured (**a**) pulse repetition rates and (**b**) pulse duration as a function of time for SA prepared using SM and PLDT.

To investigate the stability of EFDL with SAs prepared using SM and PLDT, the SAs are continuously exposed at a fixed 45.7 mW pump power for 3–5 h. The average output power, pulse duration, pulse repetition rate, and V_P–P_ data of the optical pulses generated by EDFL were continuously recorded and presented in Figs. 13, 14 and 15.

The stability of EDFL based on SAs prepared using SM and PLDT was investigated and the peak-to-peak voltage of optical pulses was recorded using an oscilloscope. First, we measured the output power for both SAs using a power meter attached to a computer for continuous 3 h but no significant difference was noticed, as both data sets shows similar behavior as function of time. The average output power at fixed pump power 45.7 mW as a function of time is shown in Fig. 13 for SAs prepared using SM and PLDT. In literature, the stability of fiber lasers is investigated by measuring the output power of optical spectrum^26–29,35^.

It seems to us (refer Fig. 13), that the observed instability in the pulsed trains was not visible in the output power. To resolve the issue of the pulse instability, we recorded V_P–P_ for SAs prepared using SM and PLDT using the oscilloscope, and the results are shown in Figs. 14 and 15. Figure 14 shows that initially the V_P–P_ is 890 nV for SA prepared using the SM technique and 1.01 mV for SA prepared using PLDT. Then a slight increase in the V_P–P_ is noticed for SA prepared using the SM approach and after 17 mins it reached its maximum value of 1.07 mV but no significant difference is noted for SA fabricated using PLDT. On achieving the optimum value of 1.07 mV, the V_P–P_ decreases up to 776 nV for the next 74 mins but for SA based on PLDT, the output voltage remains stable. Furthermore, for the next 120 mins, a rapid decrease in the V_P–P_ was noticed for the SA prepared using the SM technique and no such instability was measured for the PLDT-based SA. These results further depict that for SA prepared using the SM technique the V_P–P_ continuously decreases and finally approaches zero after 330 mins. At this point, the pulses completely disappeared for the SM-based SA whereas the V_P–P_ still remain stable for PLDT-based SA.

The comparison of measured RF linewidth and pulse width for the SAs prepared using SM and PLDT is shown in Fig. 15a,b, respectively. Initially, the repetition rates decrease, and then an increasing trend is observed and during 5 h, the repetition rates vary from 18 to 26 kHz for SA fabricated using the SM technique. However, no significant change in the repetition rates was noticed for SA prepared using PLDT. The minimum and maximum repetition rates for this SA were measured to be 21.17 and 21.57 kHz, respectively (refer to Fig. 15a). Besides, the pulse width data of EDFL based on SA prepared using SM and PLDT is further compared and shown in Fig. 15b. This data shows that for the SA prepared using the SM approach, the pulse width varies from 18 to 20 µs. However, for the SA prepared using PLDT, the minimum and maximum pulse width were noticed to be 17.2–17.9 µs, respectively. This data further suggests that PLDT provides much better stable optical pulses compared to the conventional SM technique.

The results presents in Figs. 14 and 15 confirm the stability of SAs prepared using PLDT. The better stability of SAs prepared using PLDT can be attributed to the process of fabrication of SAs. During the deposition of the thin film by PLD, the atoms/ions are deposited on the fiber ferrule with much better adhesion and without any additional impurity e.g.; PVA, etc. In PLDT, better adhesion without any additional chemicals and a direct growth of ZnO on the fiber ferrule under control environment results in better stability of SAs.

## Conclusion

In summary, we demonstrated a passively Q-switched EDFL based on ZnO-SA prepared using SM and PLDT techniques. The pulse duration, repetition rates, average output power, peak energy, pulse energy, and stability for SAs prepared using both approaches have been measured. These results suggest that for SA prepared using the SM technique, as the pump power was increased from 22 mW to 75.3 mW, the pulse repetition rates increase from 11.59 to 40.91 kHz and the pulse duration decreases from 24.91 to 10.69 µs. Besides at pump power of 75.3 mW, the peak power, pulse energy, and average output power are observed to be 0.327 mW, 2.86 nJ, and 0.18 mW, respectively. On the other hand, when PLDT-based SA was incorporated into the ring cavity, with an increase in pump power from 22.2 mW to 418 mW, the pulse repetition rates increase from 10.79 to 79.37 kHz and the pulse width decreases from 23.58 to 5.6 µs. The peak power, pulse energy, and average output power are observed to be 10.9 mW, 74 nJ, and 4.65.35 mW, respectively. Furthermore, we first measured and compared the pulse stability of EDFL based on SAs prepared using SM and PLDT for more than 5 h in terms of peak-to-peak voltage. It was revealed that for SA prepared using PLDT, the peak-to-peak voltage, pulse duration, and repetition rate remains stable for a long span of time. It can also be concluded that the stability study of EDFL would not be reliable without measuring the stability of peak-to-peak voltages of the pulsed trains. This study further suggests that PLDT is a promising technique for the fabrication of ultra-stable SAs due to their potential photonics applications.

## Data Availability

The datasets used and/or analysed during the current study available from the corresponding author on reasonable request.

## References

[1] Liu X, Han D, Sun Z, Zeng C, Lu H, Mao D, Cui Y, Wang F (2013). Versatile multi-wavelength ultrafast fiber laser mode-locked by carbon nanotubes. Sci. Rep..

[2] Addanki S, Amiri IS, Yupapin P (2018). Review of optical fibers-introduction and applications in fiber lasers. Results Phys..

[3] Fu Z, Yang D, Ye W, Kong J, Shen Y (2009). Widely tunable compact erbium-doped fiber ring laser for fiber-optic sensing applications. Opt. Laser Technol..

[4] Li D, Jussila H, Wang Y, Hu G, Albrow-Owen T, Howe R, Ren Z, Bai J, Hasan T, Sun Z (2018). Wavelength and pulse duration tunable ultrafast fiber laser mode-locked with carbon nanotubes. Sci. Rep..

[5] Li X, Wu K, Sun Z, Meng B, Wang Y, Wang Y, Yu X, Yu X, Zhang Y, Shum PP, Wang QJ (2016). Single-wall carbon nanotubes and graphene oxide-based saturable absorbers for low phase noise mode-locked fiber lasers. Sci. Rep..

[6] Ahmad H, Albaqawi HS, Yusoff N, Reduan SA, Yi CW (2020). Reduced graphene oxide-silver nanoparticles for optical pulse generation in ytterbium-and erbium-doped fiber lasers. Sci. Rep..

[7] Ahmad H, Reduan SA, Ali ZA, Ismail MA, Ruslan NE, Lee CSJ, Puteh R, Harun SW (2015). C-band Q-switched fiber laser using titanium dioxide (TiO_2_) as saturable absorber. IEEE Photonics J..

[8] Al-Hayali SKM, Mohammed DZ, Khaleel WA, Al-Janabi AH (2017). Aluminum oxide nanoparticles as saturable absorber for C-band passively Q-switched fiber laser. Appl. Opt..

[9] Shen Y, Wang Y, Luan K, Huang K, Tao M, Chen H, Yi A, Feng G, Si J (2016). Watt-level passively Q-switched heavily Er3+-doped ZBLAN fiber laser with a semiconductor saturable absorber mirror. Sci. Rep..

[10] Armas-Rivera I, Rodriguez-Morales LA, Durán-Sánchez M, Avazpour M, Carrascosa A, Silvestre E, Kuzin EA, Andrés MV, Ibarra-Escamilla B (2021). Wide wavelength-tunable passive mode-locked Erbium-doped fiber laser with a SESAM. Opt. Laser Technol..

[11] Yan P, Lin R, Ruan S, Liu A, Chen H, Zheng Y, Chen S, Guo C, Hu J (2015). A practical topological insulator saturable absorber for mode-locked fiber laser. Sci. Rep..

[12] Haris H, Harun SW, Muhammad AR, Anyi CL, Tan SJ, Ahmad F, Nor RM, Zulkepely NR, Arof H (2017). Passively Q-switched Erbium-doped and Ytterbium-doped fibre lasers with topological insulator bismuth selenide (Bi_2_Se_3_) as saturable absorber. Opt. Laser Technol..

[13] Jagadish, C. & Pearton, S. J. (eds.) *Zinc Oxide Bulk, Thin Films and Nanostructures: Processing, Properties, and Applications* (Elsevier, 2011).

[14] Janotti A, Van de Walle CG (2009). Fundamentals of zinc oxide as a semiconductor. Rep. Prog. Phys..

[15] Kumar R, Kumar G, Umar A (2014). Pulse laser deposited nanostructured ZnO thin films: A review. J. Nanosci. Nanotechnol..

[16] Ahmad H, Lee CSJ, Ismail MA, Ali ZA, Reduan SA, Ruslan NE, Ismail MF, Harun SW (2016). Zinc oxide (ZnO) nanoparticles as saturable absorber in passively Q-switched fiber laser. Opt. Commun..

[17] Johnson JC, Knutsen KP, Yan H, Law M, Zhang Y, Yang P, Saykally RJ (2004). Ultrafast carrier dynamics in single ZnO nanowire and nanoribbon lasers. Nano Lett..

[18] Khokhra R, Bharti B, Lee HN, Kumar R (2017). Visible and UV photo-detection in ZnO nanostructured thin films via simple tuning of solution method. Sci. Rep..

[19] Naharuddin NZA, Bakar MA, Sadrolhosseini AR, Tamchek N, Alresheedi MT, Abas AF, Mahdi MA (2022). Pulsed-laser-ablated gold-nanoparticles saturable absorber for mode-locked erbium-doped fiber lasers. Opt. Laser Technol..

[20] Li L, Wang Y, Wang X, Lv R, Liu S, Chen Z, Wang J (2018). Generation of dark solitons in Er-doped fiber laser based on ferroferric-oxide nanoparticles. Opt. Laser Technol..

[21] Sadeq SA, Harun SW, Al-Janabi AH (2018). Ultrashort pulse generation with an erbium-doped fiber laser ring cavity based on a copper oxide saturable absorber. Appl. Opt..

[22] Ahmad H, Ruslan NE, Ismail MA, Reduan SA, Lee CSJ, Sathiyan S, Sivabalan S, Harun SW (2016). Passively Q-switched erbium-doped fiber laser at C-band region based on WS 2 saturable absorber. Appl. Opt..

[23] Ahmad H, Suthaskumar M, Tiu ZC, Zarei A, Harun SW (2016). Q-switched Erbium-doped fiber laser using MoSe2 as saturable absorber. Opt. Laser Technol..

[24] Yan P, Lin R, Ruan S, Liu A, Chen H (2015). A 2.95 GHz, femtosecond passive harmonic mode-locked fiber laser based on evanescent field interaction with topological insulator film. Opt. Express.

[25] Asghar H, Ahmed R, Umar ZA, Baig MA (2022). A novel technique for the fabrication of a saturable absorber for fiber lasers: Pulsed laser deposition. Laser Phys. Lett..

[26] Sadeq SA, Al-Hayali SK, Harun SW, Al-Janabi A (2018). Copper oxide nanomaterial saturable absorber as a new passive Q-switcher in erbium-doped fiber laser ring cavity configuration. Results Phys..

[27] Ahmad H, Samion MZ, Kamely AA, Ismail MF (2019). Mode-locked thulium doped fiber laser with zinc oxide saturable absorber for 2 μm operation. Infrared Phys. Technol..

[28] Husin SAS, Muhammad FD, Abdullah CAC, Ribut SH, Zulkifli MZ, Mahdi MA (2019). Zinc-oxide nanoparticle-based saturable absorber deposited by simple evaporation technique for Q-switched fiber laser. Chin. Phys. B.

[29] Muhammad FD, Husin SAS, Ng EK, Lau KY, Abdullah CAC, Mahdi MA (2021). Zinc-oxide/PDMS-clad tapered fiber saturable absorber for passively mode-locked erbium-doped fiber laser. Chin. Phys. B.

[30] Anjum A, Ahmed R, Umar ZA, Azzam S, Hussain T, Sarwar MN, Baig MA (2022). Structure and defects-related optical properties of highly (002)-oriented zinc oxide thin films. Phys. B.

[31] Damen TC, Porto SPS, Tell B (1966). Raman effect in zinc oxide. Phys. Rev..

[32] Chen SJ, Liu YC, Shao CL, Mu R, Lu YM, Zhang JY, Shen DZ, Fan XW (2005). Structural and optical properties of uniform ZnO nanosheets. Adv. Mater..

[33] Ahmad H, Sharbirin AS, Ismail MF (2019). 1.8 µm passively Q-switched thulium-doped fiber laser. Opt. Laser Technol..

[34] Hu T, Hudson DD, Jackson SD (2014). Stable, self-starting, passively mode-locked fiber ring laser of the 3 μm class. Opt. Lett..

[35] Asghar H, Ahmed R, Sohail M, Umar ZA, Baig MA (2022). Q-switched pulse operation in erbium-doped fiber laser subject to CdS nanoparticles-based saturable absorber deposit directly on the fiber ferrule. Opt. Mater..

